# New opportunities in plant microbiome engineering for increasing agricultural sustainability under stressful conditions

**DOI:** 10.3389/fpls.2022.899464

**Published:** 2022-09-15

**Authors:** Muhammad Siddique Afridi, Muhammad Ammar Javed, Sher Ali, Flavio Henrique Vasconcelos De Medeiros, Baber Ali, Abdul Salam, Sumaira, Romina Alina Marc, Dalal Hussien M. Alkhalifah, Samy Selim, Gustavo Santoyo

**Affiliations:** ^1^Department of Plant Pathology, Federal University of Lavras (UFLA), Lavras, MG, Brazil; ^2^Institute of Industrial Biotechnology, Government College University, Lahore, Pakistan; ^3^Department of Food Engineering, Faculty of Animal Science and Food Engineering, University of São Paulo (USP), São Paulo, Brazil; ^4^Department of Plant Sciences, Quaid-i-Azam University, Islamabad, Pakistan; ^5^Zhejiang Key Laboratory of Crop Germplasm, Department of Agronomy, College of Agriculture and Biotechnology, Zhejiang University, Hangzhou, China; ^6^Department of Biotechnology, Quaid-i-Azam University, Islamabad, Pakistan; ^7^Food Engineering Department, Faculty of Food Science and Technology, University of Agricultural Sciences and Veterinary Medicine of Cluj-Napoca, Cluj-Napoca, Romania; ^8^Department of Biology, College of Science, Princess Nourah Bint Abdulrahman University, Riyadh, Saudi Arabia; ^9^Department of Clinical Laboratory Sciences, College of Applied Medical Sciences, Jouf University, Sakaka, Saudi Arabia; ^10^Instituto de Investigaciones Químico-Biológicas, Universidad Michoacana de San Nicolás de Hidalgo, Morelia, Mexico

**Keywords:** plant microbiome, fungi, sustainable agriculture, biotic and abiotic constraints, PGPR – plant growth-promoting rhizobacteria

## Abstract

Plant microbiome (or phytomicrobiome) engineering (PME) is an anticipated untapped alternative strategy that could be exploited for plant growth, health and productivity under different environmental conditions. It has been proven that the phytomicrobiome has crucial contributions to plant health, pathogen control and tolerance under drastic environmental (a)biotic constraints. Consistent with plant health and safety, in this article we address the fundamental role of plant microbiome and its insights in plant health and productivity. We also explore the potential of plant microbiome under environmental restrictions and the proposition of improving microbial functions that can be supportive for better plant growth and production. Understanding the crucial role of plant associated microbial communities, we propose how the associated microbial actions could be enhanced to improve plant growth-promoting mechanisms, with a particular emphasis on plant beneficial fungi. Additionally, we suggest the possible plant strategies to adapt to a harsh environment by manipulating plant microbiomes. However, our current understanding of the microbiome is still in its infancy, and the major perturbations, such as anthropocentric actions, are not fully understood. Therefore, this work highlights the importance of manipulating the beneficial plant microbiome to create more sustainable agriculture, particularly under different environmental stressors.

## Introduction

Different researchers have highlighted that by 2050, it is expected that the world population will reach 10 billion people. The massive surge in population will increase the amount of food necessary for the entire planet to be fed. However, food could be a problem for this drastically increased population. Even today, approximately 9% of the world’s population (690 million people) go to bed with an empty stomach each night (Sakschewski et al., 2014). Combining these challenges without compromising the environment and human health is a major issue in the agricultural production sector and the forefront of many plant scientists.

To achieve this goal, it will be obligatory to engage two closely associated goals. The first is to improve crop yield, especially for cereal crops, which can be accomplished through different procedures, such as genetic modification, selective breeding, avoiding waste in irrigation as well as fertilization regimes (Beddington, 2010; Godfray et al., 2010; Singh et al., 2022). Second, curtail crop losses due to pests and diseases, which have been causing losses on the order of 20–40%, in addition to the indirect effects on livelihoods and the environment (Oerke, 2006; Beddington, 2010; Godfray et al., 2010; Savary et al., 2012; McDonald and Stukenbrock, 2016).

Implementing strategies to attain the latter is challenging, particularly because the elements that corroborate plant maladies are extremely complex and multivariate (Savary et al., 2012). Moreover, cereal crops are affected by several different organisms, e.g., a variety of bacteria, fungi, oomycetes, nematodes, and viruses (Dean et al., 2012).

Fungal species competence to survive in soil mainly invade the plant roots, causing various notorious diseases in plants while simultaneously undermining the host plant of its nutrients; this is the case for wheat disease caused by *Gaeumannomyces graminis var. triciti*, which in some cases can eradicate an entire wheat crop. Thus, worldwide, the take-all of wheat is considered the most important root ailment of wheat (Coombs, 2004; Kwak and Weller, 2013; Cook et al., 2015; Hernández-Restrepo et al., 2016; Ahmad M. et al., 2022). Plant-parasitic nematodes living in the same vicinity as plant roots are among the most destructive plant pathogens, causing estimated damage of more than US$100 billion per year. An expert-based assessment of crop health listed nematodes as among the most damaging pests and pathogens for different crops (Savary et al., 2012).

To avoid crop losses due to maladies, chemical pesticides are routinely applied on crops, with the main goal of eradicating or lessening the disease invasion, infection or severity. However, it is becoming increasingly clear that long-term chemical pesticide usage poses several adverse effects on the environment and human health (Sanyal and Shrestha, 2008; Kortekamp, 2011). For instance, a myriad of pesticides can cause acute and chronic toxicity in humans, and they are progressively being shown to cause widespread damage to the broader ecosystem, affecting non-target organisms, such as pollinator species, and soil pollution and water (Arora and Sahni, 2016; Grewal et al., 2017; Anwar et al., 2023). These non-target effects can also extend to reduce the beneficial microbial diversity within soil, which in turn refrains and suppresses the available populations of pathogens from competition and elevates the risks of pathogen invasion and colonization of plant tissues (Jacobsen and Hjelmsø, 2014). Additionally, plant pathogen genetic evolution and resistance against various resistant bread crop varieties can be devastating outcomes of the continuous application of pesticides, that pathogens can rapidly evoke plant host resistance mechanisms, especially when only a single gene is responsible for resistance. In certain circumstances, there are many crop species for which resistant cultivars are unavailable. For instance, every 2–3 years, rice cultivars that are usually resistant to *M. oryzae* typically become ineffective. These combined issues have opened up ways to search for another alternative.

Plant-associated microbiomes have essential functions in improving plant nutrition acquisition and provide protection against biotic and abiotic stressors. Nutrient acquisition has been thoroughly studied for plant symbioses with arbuscular mycorrhizal fungi (AMF) and Rhizobium bacteria (Bergelson et al., 2019; Trivedi et al., 2020). Additionally, these diverse microbial communities of plant microbiome perform multiple functions such as nitrogen fixation, nutrient solubilization, (Adnan et al., 2022; Khan et al., 2023) protection against devastating plant pathogens and production of phytohormones (Haider et al., 2022) like indole acetic acid, auxin, gibberellin, abscisic acid, aminocyclopropane-1-carboxylate deaminase, antibiotics, development of induced resistance to pathogens in plants, and promotion of the population of other helpful microorganisms (Afridi et al., 2019, 2021; Mehmood et al., 2021a; Zainab et al., 2021).

Manipulation of the soil microbiome for plant growth and protection is considered one of the possible avenues in previous decades. The soil microbiome has complex interactions with the plant and its roots, helping to remove contaminants, provide nutrients, and proliferate growth (Liu et al., 2019). Continued research into this subject matter is necessary to elucidate the complex interactions that occur so that manipulating these relations may be used to help feed 10 billion people. Therefore, this review aimed to highlight the beneficial services of the plant-associated microbiome to be manipulated and optimized, resulting in better agricultural production, even under non-optimal conditions.

## Defining the plant microbiome

Plants are associated with a diverse group of microbes, such as bacteria, oomycetes, fungi, archaea, and viruses, through three major associations, the rhizosphere (root-attached soil), endosphere (internal tissue), and phyllosphere (aboveground parts), which execute significant activities that influence host health and fitness and inhabit a well-defined area of plant microbiome. Among them, the rhizosphere is the most complex and diverse niche of microbial communities (Lakshmanan et al., 2014; Bandyopadhyay et al., 2017).

Plants have evolved to form complex, beneficial relationships with the microorganisms in their surroundings. Although the plant microbiome includes bacteria, fungi, archaea, protists and viruses, the majority of research has focused on bacterial and fungal communities (Trivedi et al., 2020). These organisms play important roles in the health and productivity of crops by forming complex co-association with plants (Fitzpatrick et al., 2018). In particular, plant-associated microbiota and plants form a ‘holobiont,’ and evolutionary selection among microbes and plants contributes to the stability of the ecosystem (Hamonts et al., 2018; Xu et al., 2018a).

Recently, developed culture-independent high-throughput sequencing has accelerated the identification of microbial communities inhabiting the surrounding spaces, as well as inside tissues and surfaces of plants, and demonstrated the existence of microbial lineage subsets, termed ‘core microbiota,’ which reproducibly make contacts with host plants across a wide range of environmental conditions (Bergelson et al., 2019).

**FIGURE 1:**
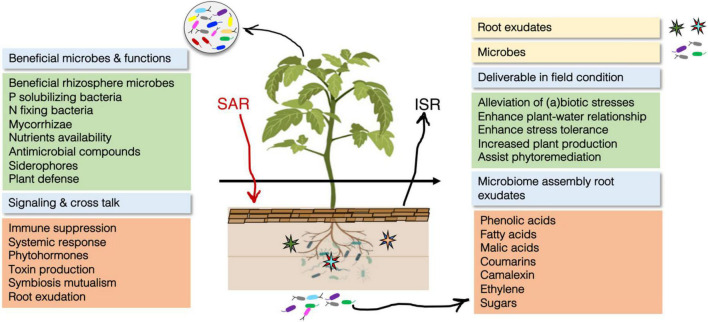
The holistic overview of plant microbiome compositions, the interaction between plant and its associated microbes, function and its positive effect on plant growth and development under extreme conditions. Plant recruit and assembly beneficial microbes via exudation and constitute a healthy and beneficial microbial community. This microbiome improves plant health, alleviates abiotic stresses and provides a safeguard to the host exhibiting various direct and indirect mechanisms.

In terms of therapeutic or diagnostic benefits and technical advancements, the study of the microbial community has been a leading interest amongst scientific society. In addition to compensation, all or some of these microbes actively support plant improvement (Parray and Shameem, 2019). In accordance with distribution, these microbes can be found in the phyllosphere (above the ground–stem and tissues), endosphere (underground–tissues within the plant) and rhizosphere (roots alongside growth layers) of the host (Figure 1). This is because the plant anatomy represents and provides a remarkably suitable environment for these microbes (Schlaeppi and Bulgarelli, 2015). Over the past decades, individual microbes from these microbiomes have displayed exceptional features (Rani et al., 2019; Gupta et al., 2020) containing their interactions with the host. The symbiotic association has been determined to be pathogenic and/or non-pathogenic to the host plants, including nitrogen fixation, development, bioremediation and stress tolerability (Chhabra and Dowling, 2017; Roth and Paszkowski, 2017; Li et al., 2019). To overview an extended mutualistic to parasitic and commensalism dealing, plants correlated with the microbiota cover a large portion. Additionally, the study of this connection may lead to in-depth knowledge and could provide appreciative outputs.

According to the growth of the global population, a sustainable environment of high food security is urgently needed, which is achieved mostly by strengthening crop practices. In this regard, the microbial system has been a key technology in such progress. Since ∼300 BC, this goal has been founded by the manipulation of the soil microbiome (Vessey, 2003), which is a key to the green revolution (Parnell et al., 2016). It is interesting to note that soil microbiomes are now touted as a cornerstone of the next green revolution.

## The plant microbiome at work

The microbiome, as a ‘second genome’ of organisms, including plants, has a mutualistic relation with health and general well-being. Taking this into consideration, Figure 1 depicts a holistic overview of the plant microbiome with some attributes, signaling and cross-talk between the plant and its relevant biota. This mutualism can be direct and/or indirect; plant–plant, microbe–microbe, plant–microbe, and/or with some microbe–microbe and macrosoil eukaryote interactions (Tarkka et al., 2008). In addition, these interactions could be classified into competition, parasitism, mutualism and commensalism. Being more common, the latter two interactions provide major benefits to one or both interacting species.

### Microbial services

Within this context, the manipulation of the phytomicrobiome can be of greater interest to boost diagnostics and therapies in plants, which are extendable to animals and humans in the future (Zmora et al., 2016). However, the phytomicrobiome is generally associated with multiple microorganisms that are major factors for agricultural production and play a critical function. Agricultural sustainability has been a major proposal in the world and has been completed by the implementation of many microorganisms. In fact, some of these microbes colonized the plant roots, improve plant growth and regulate vital functions against detrimental pathogens and thereby lead to plant productivity (van der Heijden and Hartmann, 2016; Cordovez et al., 2019; Rafique et al., 2019). The world is transitioning to ecologically safe and economically effective approaches that could be used to promote agricultural productivity. Therefore, a balanced farming system is critical in terms of the survival of Earth. In this regard, crop output per unit area of land must be raised to fulfill the demand for food (Doran, 2002). As per recommendations, an equivalent improvement in plant health could be achieved via various strategies. Among them, PGPR, as probiotics for plant roots and prebiotic substrates/additives, can be used to cause compositional alterations in the phytomicrobiome and are termed soil amendments. The plant microbiome has a strong influence on nutrient availability and the growth and development of the host (Carvalhais et al., 2013). Accordingly, plants on the basis of natural exudate recruit and “engineer” a local microbiome (Kumar et al., 2018b; Rojas-Solís et al., 2018) and make this habitat fit to their survival.

### Signaling and cross-talk

In general, plants of the local habitat are in cross-talk with numerous surrounding stimuli incorporating the microbial communities (Figure 1); therefore, this is termed the homeostatic photomicrobiome. In such a homeostatic phytomicrobiome, plants are allowed to sense and properly respond to any interactive stimulus of the system. However, after microbial substance recognition, they can ultimately lead to mutualism or immunity. Furthermore, communicatory signaling is an important phenomenon responsible for healthy lifestyles and the survival of organisms (Cook et al., 2015; Müller et al., 2016). This communicatory network can be predicted for any of the micro- or macroorganisms living on the planet, such as quorum sensing bacteria (Cornforth et al., 2014), whales (Parks et al., 2015) and those across the tree of life. Such communicating circuitry plays a decisive role in the evolution of the life of associated organisms (West et al., 2015). Overlooking such a communicatory web, chemical signaling is highly vital and participates in perception and modulation in stationary organisms, such as plants. However, plants use these chemical bases as signals to maintain mutual links with presided microbes either on the aerial (trunk, shoots, leaves, etc.) and/or the underground parts (roots). As per estimation, approximately 5–20% photosynthetically fixed carbon has been an active ingredient in plant rhizosphere-inducing microbes for healthier microbial community formation (Marschner, 1995). In addition to carbon, microorganisms discharge many more signaling chemical substances to the rhizosphere. Through them, the most prominent are phytohormones, extracellular enzymes, organic acids, antibiotics, volatile contents and surface factors, e.g., immunomodulatory precursors such as flagellins and lipopolysaccharides in Pseudomonas (Ping, 2004; Dangl et al., 2013). As a signaling molecule, quorum sensing, e.g., *N*-acyl-homoserine lactones (AHLs), when secreted, is used to regulate gene expression by plant-associated bacteria (Berendsen et al., 2012). However, AHLs have been major precursors affecting root development in a model plant of Arabidopsis (Ortíz-Castro et al., 2008). Moreover, AHLs have the tendency to elicit “systemic resistance” (ISR) that allows plants to evade lethal pathogens without requiring bacterial factors. This effect can be a systemic mechanism because the roots are inoculated with manifold plant growth-promoting rhizobacteria (PGPR), such as *Pseudomonas*, *Burkholderia*, and *Bacillus* sp. that turn host plants non-susceptible to invaders (Schuhegger et al., 2006; Choudhary et al., 2007; Tarkka et al., 2008). In line, such a microbial combination is essential and responsible for fitness and plant health and beyond fulfilling fundamental demands (water, nutrients, etc.), they increase the tolerability of plants against any of the (a)biotic stressors (van der Heijden and Hartmann, 2016; Cordovez et al., 2019). This association provides the main benefits to soil biochemistry to suppress soil-borne diseases and detrimental pathogens. It is noteworthy that these pathogens may still be present but in an inactive state that would not be able to cause soil-borne diseases or damage their resident host, while this setup can be termed “soil suppression.”

## The relevant role of plant-associated fungi and bacteria

Plants can be associated with an immense diversity of microorganisms, including fungi. There is sufficient evidence that some fungi, such as AMF, can provide broad benefits to the plant in a type of symbiotic interaction. AMF are obligate biotrophic organisms that supply mineral nutrients to the host plant and, in return, receive carbon derived from photosynthesis. In this same sense, AMF can modulate carbon distribution in plants by modifying the expression and activity of key enzymes for the synthesis, transport and/or catabolism of carbon compounds, such as sucrose. Since sucrose can be essential for the maintenance of all metabolic and physiological processes, the modifications addressed by AMF can significantly affect plant development and responses to stress. Additionally, the interaction between AMF and plants can also host lipid biosynthesis to acquire storage reserves and generate biomass.

Other fungal species that provide various services to the plant are *Trichoderma* spp. richoderma (teleomorph Hypocrea) is a fungal genus that inhabits many ecosystems, including those involved in agricultural and production practices. There are several examples of how Trichoderma is part of microbial bioinoculants, either individually or carrying out synergistic interactions with other microorganisms, such as plant growth-promoting bacteria or PGPB. *Trichoderma* species, such as *T. harzianum*, *T. viride*, and *T. virens*, among many more species, can ameliorate the severity of plant diseases by inhibiting the growth of phytopathogens in the soil (mainly), since they exhibit antagonistic and mycoparasitic activities. Additionally, it has been reported that *Trichoderma* spp. It is also capable of interacting directly with the roots, which leads to promoting the growth and development of vegetable crops, as well as of course, stimulating resistance to diseases and tolerance to multiple types of environmental stress, such as salinity or drought to name a few. To further explore topics on the importance of plant-associated fungi and their beneficial role, readers are directed literature (Santoyo et al., 2021).

The root surfaces tightly adhering to the rhizosphere’s soil interface colonize these PGPR (Jain, 2016). PGPR-mediated biocontrol processes are wide-ranging, like availability of nutrients and ecological niches, synthesis of allelochemicals including enzymes and antibiotics, development of induced resistance to pathogens in plants, and promotion of the population of other helpful microorganisms (Table 1). The best-known PGPR that colonizes in the rhizosphere strains are *Bacillus, Rhizobium, Acinetobacter, Alcaligenes, Arthrobacter, Enterobacter, Pseudomonas, Serratia*, and *Burkholderia* (Vinayarani and Prakash, 2018; Mehmood et al., 2021b) successfully induce disease resistance against the bacterial pathogen in plants, including *R. solanacearum* (Cao et al., 2018), *E. carotovora* (Chandrasekaran and Chun, 2016), *D. solani*, *E. amylovora*, and *P. carotovorum* (Vega et al., 2019). Both growth promotion and biological control can regulate by the same strain of PGPR. Generally, biological control of these bacteria relies on direct or indirect modes of action; however, all these mechanisms are highly influenced by the type of host plants (Dey et al., 2004; Singh et al., 2019). In direct mechanism, pathogens directly affected by the production of metabolites, for instance, antibiotics, hydrogen cyanide (HCN), iron-chelating siderophores, pyoluteorin, tensin, 2,4- diacetylphloroglucinol, phenazines, viscosinamide, and other cell wall-degrading enzymes, while another mechanism is known as induced systemic resistance, this happens by the intervention of an inducing agent that systemically stimulates the chemical or physical defensive mechanisms of the host plant, resulting in decreased symptoms of pathogens that invade tissues distal to the inducer (Table 1; Khatoon et al., 2020; Raj et al., 2020).

**TABLE 1:** Plant growth promoting microbes underpinning plant growth and enhance tolerance against biotic and abiotic stresses employing various mechanisms.

Host species	PGPR	Functions/Response	References
*Arabidopsis thaliana*	*B. phytofirmans* PsJN	Abscisic acid signaling, proline and ROS production	Pinedo et al., 2015
*Arabidopsis thaliana*	*B. subtilis* GB03	Import of Sodium ions in root	Wang et al., 2016
*Arabidopsis thaliana*	*P. yonginensis* DCY84T	ROS Detoxification, Sodium ion homeostasis	Sukweenadhi et al., 2015
*Abelmoschus esculentus*	*Enterobacter* sp. UPMR18	ROS pathway Antioxidant enzymes production	Habib et al., 2016
*Glycine max*	*P. simiae strain* AU	Antioxidant enzymes Production	Vaishnav et al., 2016
*Glycine max*	*B. firmus* SW5	Production of antioxidant enzymes, salinity tolerance,	El-Esawi et al., 2018
*Gossypium hirsutum*	*Brucella* sp. PS4	Pesticide degradation	Ahmad S. et al., 2022
*Puccinellia tenuiflora*	*B. subtilis* GB03	Modulation of Na^+^ homeostasis	Niu et al., 2016
*Saccharum officinarum*	*B. xiamenensis*	Phytoremediation	Zainab et al., 2021
*Solanum lycopersicum*	*B. megaterium*	Metallothionein Glutathione reductase enzyme synthesis	Zameer et al., 2016
*Solanum lycopersicum*	*E. cloacae* PM23	ROS Detoxification, Sodium ion homeostasis	Ali et al., 2022b
*Solanum lycopersicum*	*B. safensis* (SCAL1)	Heat Stress	Mukhtar et al., 2022
*Solanum lycopersicum*	*B. anthracis* PM21	Phytoremediation	Ali et al., 2021
*Solanum tuberosum*	*B. subtilis* PM32	Fungal diseases biocontrol	Mehmood et al. (2021a)
*Solanum tuberosum*	*B. mycoides* PM35	Proline production, and ROS scavenging	Ali et al., 2022a;
*Solanum lycopersicum* L.	*B. safensis* Strain SCAL1	Produced exopolysaccharide and ACC deaminase	Mukhtar et al., 2022
*Zea mays* L.	*B. amyloliquefaciens* SQR9	Photosynthesis, Na^+^ export, and sequestration	Chen L. et al., 2016
*Lettuce microcosms*	*T. hamatum GD12*	*N-acetyl*-β*-D-*glucosaminidase genes	Ryder et al., 2012
*Curcuma longa* L	*T. harzianum* TharDOB-31	Indole-3-acetic acid hydrogen cyanide production	Vinayarani and Prakash, 2018
*Solanum lycopersicum*	*A. pullulans 490*	Produces biosurfactants, biocontrol activity	Köhl et al., 2020
*Solanum lycopersicum*	*C. rosea 016*	Produces biosurfactants, biocontrol activity	Köhl et al., 2020
*Capsicum annuum L*	*Beauveria bassiana*	Niche or resources and antibiosis	Jaber and Alananbeh, 2018
*Pinus radiata*	*F. circinatum*	Antagonism,	Martínez-Álvarez et al., 2016
*Poncirus trifoliata*	*F. mosseae*	Drought stress, Hyphal water absorption rate	Zhang et al., 2018
*Triticum aestivum*	*G. mosseae*	Drought stress, osmotic potential, antioxidant enzymes	Rani, 2016
*Triticum aestivum* L.	*R. irregularis*	Heat stress, nutrient allocation nutrient composition in root	Cabral et al., 2016
*Zea mays*	*R. intraradices*	High temperature, enhanced transpiration photosynthetic rate	Mathur et al., 2016
*Solanum lycopersicum*	*R. irregulari*	High temperature, Enhanced photosynthetic phosphorylation	Calvo-Polanco et al., 2016
*Cucumis sativus L.*	*G. intraradices*	Salinity stress, enhanced antioxidant enzymes, biomass	Hashem et al., 2018
*Solanum lycopersicum* L.	*R. irregularis*	Salinity stress, Enhanced biomass and growth hormones	Khalloufi et al., 2017

## Why engineer the plant microbiome?

In light of the intensification of cropping practices and changing climatic conditions, nourishing a growing global population requires optimizing environmental sustainability and reducing the ecosystem impacts of food production. The use of microbiological systems to ameliorate agricultural production in a sustainable and eco-friendly way is widely accepted as a future key technology. The manipulation of soil microbiomes to optimize crop productivity is an ancient practice; records can be traced to ∼ 300 BC (Vessey, 2003). It is interesting to note that soil microbiomes are now touted as a cornerstone of the next green revolution (Parnell et al., 2016). In addition, the continuous growth of the world population demands that the global availability of food be one of the major concerns in the near future. According to the projected data (DESA, 2019), if this increment continues, in turn, the demands for food will reciprocally increase by 8.5 billion in 2030, 9.7 billion in 2050, and 11 billion by 2100. However, the fulfillment of such demand must be ascertained with green and innovative technologies incorporating plant and microbial resources.

Environmental stressors have caused major alterations in plant physiology and biochemistry that lead to significant reductions in plant yield and production. In accordance with previous reports (Kumar et al., 2018a), 30–50% of agricultural losses have been impacted by unfavorable environmental conditions. Agronomic loss coupled with continual population growth demands at least a 60% boost in agrarian production to meet food demand on a larger scale (Wild, 2003). Often, the agricultural production has mostly been supplemented with pesticides. Consistently, approximately 2 million tons of pesticides are globally administered to reduce causative pests, aiming for maximum crop production (Foong et al., 2020). Concurrently, the use of agrochemicals influences biodiversity and soil fertility, biochemistry, agricultural sustainability, food safety and nutritional security, among others. However, excessive use of pesticides not only produces environmental pollution, but over time, their drastic chemical substances can cause diseases in humans and livestock (Sharma et al., 2010; Fu et al., 2022). Additionally, they kill beneficial microbes and reduce nutrient availability, which are essential elements for plant growth and productivity (Meena et al., 2020).

Thus, the plant microbiome contributes to the basic functions of microbial ecosystem services in agriculture, plant production and performance, nutrition, improved quality of the soil, and tolerance to (a)biotic stresses (Figure 1; Quiza et al., 2015; Vandenkoornhuyse et al., 2015; Enebe and Babalola, 2018; Ojuederie et al., 2019). The plant microbiome supports plants through the mechanisms of regulating hormones, specific antagonistic metabolite (rhizobitoxine) production that induces resistance against drastic pathogens, suppression of soil-borne disease, antibiosis, and competition for nutrients in the rhizosphere (Choudhary et al., 2007; Penton et al., 2014; Reitz et al., 2015; Zhang et al., 2019; Rodríguez et al., 2020).

**FIGURE 2:**
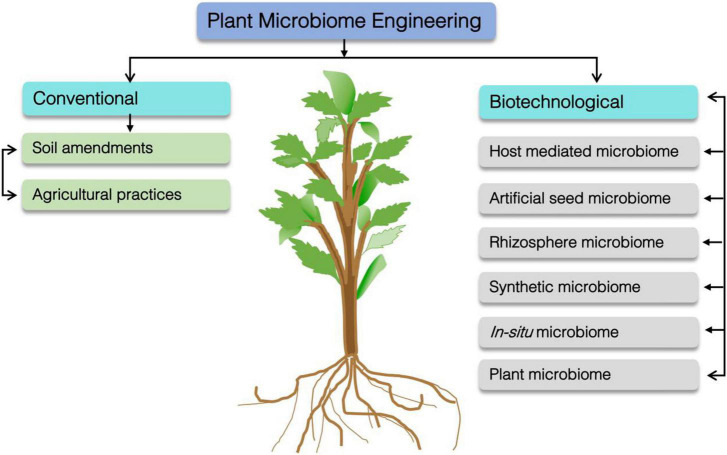
Plant microbiome engineering via biotechnological and conventional approaches. Host-mediated microbiome (indirectly selection of microbiome through utilization of host phenotype), artificial seed microbiome (artificial selection of microbiome and its integration/inoculation with seeds. This establish microbiome may evolve during the development and germination that consequently impact plant microbiome structure and function), Rhizosphere microbiome (bacterial competitiveness engineering) Synthetic microbiome (genetically engineered microbes inoculation to host plant) *In situ* microbiome (manipulation of native microbial community in their native context) Plant mycobiome (optimization and improvement of beneficial plant–fungal interactions).

Therefore, plant microbiome engineering is an alternative but an untapped strategy that can be exploited for plant health, growth, and productivity under extreme conditions. Recently, a number of accessible approaches have been proposed for plant microbiome engineering (Figure 2; Arif et al., 2020; Kumar and Dubey, 2020). An interesting avenue is to harness variations in exudation patterns to enhance the beneficial rhizosphere microbiome (Quiza et al., 2015). The microbiome can be engineered by traditionally amending soil with (in)organic supplementation and agricultural practices to promote microbial diversity, functions and interactions with the targeted host (Figure 2; Sankar Ganesh et al., 2017; Saeid and Chojnacka, 2019). Therefore, the living components of the rhizosphere can be engineered to promote plant health and growth, two features that strongly depend upon the interactions of living organisms with their environment (Dessaux et al., 2016). Thus, aiming at viable agronomic production, several innovative tools could play a central role by improving microbial bioengineering that is beneficial to replace lethal agrochemical substances.

## Engineering the plant microbiome for green agricultural production

In addition to protection, plant microbiomes provide key benefits regarding better health, with improved growth and production and plant environmental adaptation (Haney et al., 2015; Berg et al., 2016). Most microorganisms are found in such a biome that they tend to cause physiological alterations and allow plants to survive detrimental invasions (Dubey et al., 2019; Santoyo et al., 2021). Within the microbiome, these microbes are clustered on the surface and tissues of the host plants. The bimodal association thereby allows nutrient acquisition, promoting the growth and resilience of the host against environmental stressors (van der Heijden and Hartmann, 2016; Cordovez et al., 2019).

The traits displayed by the microbiome community are of high relevance to plant health, yet they are influenced by microbial diversity, unwanted conditions and even host plant species (Jain et al., 2020). The entire microbiome is not involved in corresponding functions; however, they are performed by unique microbial species because of synergistic effects between two or more strains (Rojas-Solís et al., 2018). The manipulation of the bacterial microbiome and the production of bioinoculants have enabled scientists to control and properly monitor plant health and production (Adesemoye et al., 2009).

In this regard, several strategies, including soil amendment, artificial microbial consortia and host-dependent microbiome engineering, have been proposed that could strengthen stress tolerance, disease resistance and nutrient acquisition in host plants (Figure 2) A traditional method of soil engineering or amendments is adding (in)organic substances directly to soil or using alternative agricultural tools. Any of these sources guide farmers to manipulate plant–microbiome interactions to increase crop production (Wang et al., 2015; Sankar Ganesh et al., 2017).

Conforming reported data, a host-mediated microbiome engineering approach is a host-based indirect selection of proper microbes and leveraging out those that are influential to the microbiome in context (Mueller and Sachs, 2015). In addition, an “artificial microbial consortium (AMC)” has also been used in microbiome engineering.

A recent example of biostimulant consortium application in phytomicrobiome for enhancement productivity of chickpea and soil health was conducted by Mukherjee et al. (2022). These experiments were carried out in two different locations like Banaras Hindu University Varanasi, and Sarai Dangri village, Uttar Pradesh, India. Microbial strains BHUJPCS-15 and BHUJPVCRS-1 were isolated from chickpea seed and chickpea rhizosphere soil respectively. This study depicts that consortium significantly increased yield NPK, microbial counts and soil enzymes. Interestingly, the results showed that microbiome manipulation via potential biostimulant consortium directly influenced the yields and soil health. Recently Glick and Gamalero (2021) explored in their article that mostly plant attract and beneficial microbes. This study further highlighted that bacterial consortia assist plants in various ways such as promoting plant growth and providing protection to hosts from a wide range of direct and indirect environmental stresses. This study also suggests that the microbiome could be engineered by engineering plant seeds to contain desired bacterial strains. It is unquestioned that Phytomicrobiome is an untapped source which might be potentially resolved the current and future challenges of sustainable agriculture and food security. But at the same time biotic and abiotic constraints substantially imbalance the functionality of phytomicrobiome and we are unable to overlook them (Chouhan et al., 2021). This study also recommends and shaded light on the potential of Culturable PGPR and endophytes that could be harnessed for resilient microbiome engineering.

However, in this functional consortium, an established complex interactive network of different microbes in the rhizosphere environment has been essential (Kumar et al., 2018b). Other than the rhizosphere, microbes can also be found in the root part that permits only useful microbes to access plants as endophytes (Rojas-Solís et al., 2018). As a key benefit, AMC via microbiome engineering can be used to modify the respective phytomicrobiome. An ideal AMC fabrication is based on a systematic method that can contain a series of crucial steps. Similarly, active microbe selection and regulation of their mutual interactions, excavation along the culturing core microbiota to evaluate consortium efficacy (Kong et al., 2018), are major parts of the process utilized in AMC production (Figure 2).

Additionally, genotype-dependent host microbiome engineering has been harnessed for microbiome engineering to enhance host functions and induce resistance in diverse environments. The genetic bases of plants are fundamental for the shaping and functioning of microcosms (Arif et al., 2020), such as *Pseudomonas simiae* WCS417r, for improved biomass production in Arabidopsis (Wintermans et al., 2016). This indicates a genetic relation of Arabidopsis loci (controlling plant defense and cell wall integrity) with phyllospheric bacteria (Horton et al., 2014). It has also been proven that plants can expel bacterial species into the rhizosphere, but the mechanisms by which useful or harmful microbes exchange with related holobionts are unknown.

### Improving plant growth-promoting mechanisms

The microbiome is composed of several different types of organisms, including bacteria, fungi, protozoa, archaea, and viruses (Mueller and Sachs, 2015). This array of microbial communities plays a pivotal role in the functioning of plants by influencing their physiology and development (Mendes et al., 2013). Plant microbiomes can play a beneficial role, protecting the plant from potential pathogens, improving plant growth and fitness and inducing tolerance to abiotic stresses (Haney et al., 2015; Berg et al., 2016).

Unsurprisingly, the rhizosphere microbiome also inherits soil-borne plant pathogens that colonize plant roots and successfully hack plant innate immunity by breaking the preventive microbial shield of beneficial microbes and causing disease (Mendes et al., 2013). However, it has been proven in various studies that plants secrete small molecules for the recruitment of actively beneficial microflora to assist their conformation under extreme conditions (Busby et al., 2014). It is well known that plants and associated microbes establish symbiotic relationships that facilitate nutrient acquisition and induce resistance in unfavorable environments. However, the plant unable to distinguish beneficial microbes and restrict the formation of pathogenic associations is still unknown (Zipfel and Oldroyd, 2017).

It is well documented that the interactions between plants and their microbiomes are mediated by metabolic signaling. Plant release 20–35% photosynthetic carbon into the rhizosphere in the form of metabolites that recruit beneficial microbes (Figure 1). These microbes symbiotically associate with host plants and underpin them under adverse conditions (Arif et al., 2020; Trivedi et al., 2020). However, concomitantly, the rhizosphere is also a playground and battlefield for soil-borne pathogens that establish parasitic relationships with host plants. Moreover, the diversity and population ratio of plant pathogens and beneficial microorganisms are linked to the amount and quality of plant root exudates and microbial interactions in the rhizosphere (Somers et al., 2004; Raaijmakers et al., 2009). For example, the model plant *Sorghum bicolor* secretes specific metabolites, which facilitates bacterial ATP-binding cassette transporter gene expression and, in turn, modifies the root-associated microbiome composition by promoting the abundance and activity of monoderm bacteria, which has a positive impact on the growth and development of *Sorghum bicolor* plants facing drought stress (Xu et al., 2018b). This is a potential blueprint for developing SynComs from such plant-associated microbiomes to increase crop productivity in arid areas with low precipitation and poor irrigation systems. Understanding the substantial role of metabolites and biotechnological approaches might help to unravel the mechanisms underlying beneficial microbe recruitment for microbiome engineering.

### Enhancing phytoremediation activities

Phytoremediation is an environmentally friendly, solar-powered and cost-effective soil remediation technology. Based on plant ability, this technology has to do with the already existing contamination in the system biome, where it intercepts, takes up, accumulates and translocates contaminants (Pilon-Smits, 2005). The efficiency of phytoremediation depends on plants (Vangronsveld et al., 2009), contaminant concentration, soil pH, nutrients and oxidoreduction (Sessitsch et al., 2013) as well as those microorganisms that are associated with soil and plants, respectively. Phytoremediation, instead of a better technology, has often been observed with non-uniform results at the field scale, slow and incomplete degradation, and long clean-up processes (Vangronsveld et al., 2009; Stephenson and Black, 2014). To date, the improvement in soil, contaminant availability and accessibility (de La Torre-Roche et al., 2012), plant growth (Sessitsch et al., 2013), and exploration for the exploitation of soil and plant-associated organisms in phytoremediation (Barac et al., 2004; Abhilash et al., 2012) have been main topics of interest. In recent decades, many approaches have been focused on individual organisms rather than on integrated meta-organisms, while in such regards, the potential impact has been limited. Improved phytoremediation necessitates a central understanding of plant–microbe interactions, and responses to pollutants can be of high relevance. In line, the comprehension of how the host combines the beneficial microbiome and its function under contaminant stress is unavoidable. Molecular data and ecological models in this regard have clarified the assemblage of fewer insects (Scheuring and Yu, 2012), respectively.

Beyond plants and related microorganisms, the metaorganism has shown successful improvement in agriculture practices (Mendes et al., 2013; Berg et al., 2014) and disease mitigation (Berendsen et al., 2012) and has uncovered mutual interactions between plants and unlimited degradative microbial taxa. It has been declared that the plant microbiome can be helpful in extending the functional potential of targeted hosts. Therefore, such a microbiome enables regulation of the expression of traits in plants, thus strengthening physiological state and tolerance (Mendes et al., 2013). However, it can be emphasized that the phytoremediation is microbiome dependent. Moreover, it is accepted that hosts assemble non-random sets of microbial symbionts with a higher proportion of beneficial microbes than expected. With respect to polluted soil, a host plant is free to choose microbes with degradative genes within a pool of candidates in bulk soil (Siciliano et al., 2001), but a full understanding of how hosts carry the process is lacking. Expressively, hosts can be found with a mutualistic symbiosis of PGPR and mycorrhizal fungi. Within this symbiotic association, plants provide root exudates and produce a microbial habitat, while PGPR degradative bacteria and mycorrhiza sponsor plant growth and detoxify the environment. In the presence of contamination, the rhizosphere and root microbial communities are strongly damaged (Siciliano et al., 2001).

### Ameliorating plant stress

The plant microbiome presents a complex interrelationship among many environmental factors and bacterial communities. In particular, under open field conditions, the possible bias in laboratory experiments is emphasized due to the lack of variability in environmental changes. Extreme environmental stresses, mainly climatic changes, can influence microbial communities. The soil microbiome can be affected by these stresses directly from drought-, salt- or heat-tolerant taxa (Martiny et al., 2017; Naylor et al., 2017) and indirectly by altering soil chemistry or diffusion rates (Liptzin et al., 2011).

The impact of salinity can be alleviated by the implementation of halo-tolerant synthetic microbiomes in saline soil systems. As the majority of microbes are halo-sensitive, some halophytic plant-associated members are halotolerant and can be considered potential targets for developing synthetic microbiomes. It has been demonstrated that inoculated halotolerant rhizobacteria improve the native microbial community’s resilience to salinity stress and, as a result, can improve plant growth and stability in saline states (Bharti et al., 2015; Ali et al., 2022c). An engineered microbiome approach is recommended for use in areas with saline water irrigation systems.

Drought is among the worst obstacles to agricultural productivity. Plant stress tolerance must be improved to allow acceptable crop production in limited resources of water under drought situations (Liu et al., 2019; Salam et al., 2022). Drought stress tolerance in plants based on root-associated bacteria has also been reported. In addition, molecular compositions (such as root exudates) have shown promising potential in the relevant scenario of plant microbiome perturbations. Studies have better explored an example of the biosynthetic salicylic acid in *A. thaliana* that collects a normal root microbiome (Lundberg et al., 2012). This study has shown that central regulators in the immune system of plants have an impact on root microbiome composition. Moreover, such regulators can be adapted to amend the microbial community, which, in addition to improved productivity, can increase resilience against unwanted stressors. Most studies on the plant microbiome have considered model plants, particularly *A. thaliana*. All information attained could be extrapolated to other plant communities. Therefore, more effort should be directed to microbiome engineering to enhance crop characteristics, such as tolerance against drought and diseases, thus allowing sustainable agricultural production (Dola et al., 2022). However, this technology has recently demonstrated its potential for the root microbiome of *S. bicolor*, for which drought conditions have caused the enrichment of a set of root microbes. Drought-based induced upgradation with metabolic shift was observed for the plants and microbes, revealing it to be a potential blueprint in handling the microbiome to strengthen crop fitness and upsurge production (Xu et al., 2018a).

### Stimulating antagonistic and biocontrol activities

Plant diseases are the cause of major economic losses for farmers worldwide. The FAO estimated that pests and diseases are responsible for approximately 25% of crop loss (Dean et al., 2005). There are regional differences reported: it is estimated that diseases typically reduce crop yields by 10% every year in more developed countries, but yield loss due to diseases often exceeds 20% in less developed areas. To avoid crop losses due to maladies, chemical pesticides are routinely applied on crops, with the main goal of eradicating or lessening the disease invasion, infection, or severity (McMichael et al., 2007). However, it is becoming increasingly clear that long-term chemical pesticide usage poses several adverse effects on the environment and human health (Rani et al., 2021).

Plants harbor a diverse array of microbes in the rhizosphere that establish beneficial relationships with their hosts, guarding from plant pathogens and influencing their health and fitness through direct and indirect mechanisms. Competition, hyperparasitism, antibiosis, production of extracellular enzymes, and induction of resistance are well documented mechanisms (Figure 1; Raymaekers et al., 2020). All these beneficial microbes associated with the roots of crop plants exert beneficial effects on their hosts and are referred to as plant growth-promoting biocontrol agents. Various studies have proven that plants secrete small molecules for the recruitment of actively beneficial microflora to assist their conformation under extreme conditions (Busby et al., 2014). This array of microbes possesses various biological control traits, such as competition for food space and colonization (Hunziker et al., 2015; Lloyd and Allen, 2015; Santhanam et al., 2015), antibiosis (Gómez Expósito et al., 2017), hyperparasitism (McNeely et al., 2017) and the production of degradative enzymes. In addition, these microorganisms associated with plants form a mutual association that impacts the host plant-associated microbiome and hosts an innumerable wealth of bacterial taxa, many of which promote tolerance to abiotic and biotic stresses and plant growth, suppress plant diseases, degrade xenobiotic compounds, and positively affect yields (Berg et al., 2016). This immense microbial diversity can be a target of manipulation by employing artificial microbial consortia, providing new synergistic opportunities for enhancing disease management (Poudel et al., 2016).

## Current challenges

### Difficulties in isolating and characterizing microbiomes

Firmicutes, Bacteroidetes, Proteobacteria, and Actinobacteria are the major rhizobacterial phyla that are compliant with cultivation. Several studies have been conducted for their isolation, genome sequencing and characterization of their phenotypes (Bai et al., 2015; Mauchline et al., 2015; Levy et al., 2018). Experiments are performed in laboratories mimicking their natural interaction with plants to find the key features of plant–microbe relations. These studies enable scientists to understand the microbial recruitment behavior in the rhizosphere as microorganisms take part in the growth and tolerance of the plant (Bai et al., 2015; Niu et al., 2017). Isolation makes the assembly and sequencing of individual genomes simpler. Moreover, it provides more resolved data compared to assembling metagenomes. Furthermore, the isolation step also confirmed the presence of isolates in the rhizospheric community and their interaction with the host plant (Levy et al., 2018). After isolation, strains can be easily detected for key enzymes and molecular mechanisms involved, e.g., the proteomic or transcriptomic response of a single fungus or bacterium to nutrient stress or the plant microbiome enlightens the plant growth promoting (PGP) potential of microorganisms. This helps discover novel traits of the microorganisms related to their PGP activities (Bruto et al., 2014; Lidbury et al., 2016). The phenotypes embarked with the plants for PGP traits are not revealed by *in vitro* screening methods. In this regard, fast and large-scale screening can be performed by genome sequencing, which also encourages the discovery of novel PGP traits or genes (Finkel et al., 2017). The knowledge of interactions between plants and microorganisms and the role of PGP traits or genes in enabling these interactions can be improved by combining these strategies with complementary molecular approaches, i.e., bioreporter and mutagenic expression systems (Wetmore et al., 2015).

### Efforts to assign functions to microbes

The task of assigning a specific function to an individual microbiome or a group of microbiomes is often challenging, as a completely different lifestyle is evident in species of even a particular genus. It varies from mutualist to pathogen and vice versa depending upon the transfer of functional genes between distantly related species or the environmental conditions (Qiu et al., 2009; Hacquard et al., 2016). The desired traits, such as phosphate mobilization in microbial phenotypes, are altered by this changeability (Lidbury et al., 2016). Therefore, there is a need to find more sensitive methods for the characterization of bacterial species beyond the genus level, and large-scale throughput methods are required for better functional characterization of each species (Schlaeppi and Bulgarelli, 2015). Advancement in technology, combined with modeling/computational techniques, can be very auspicious. For example, a combination of metagenomics products with the environment i.e., the adaptation of metagenomics to metaphenomics takes into account all the parameters that may sway the plant–microbiome interaction within a community or environment (Jansson and Hofmockel, 2018). This transition makes metagenomics more powerful and widens its functional capabilities, such as carbohydrate utilization or secondary metabolite production (Bulgarelli et al., 2015). Moreover, these new advancements also enable researchers to gain more specific insights into the specific taxa responsible for imparting key functional characteristics. Ready-to-use commercial kits facilitate DNA extraction from a sample easily (Prosser, 2015).

In soils, most of the microbial biomass (>90%) is dormant or inactive (Fierer, 2017), but in the rhizosphere, this number drops significantly as most of the microorganisms are made metabolically active in these habitats by plant-mediated factors (Bulgarelli et al., 2013). Microorganisms from these niches have been isolated, and their RNA is extracted to identify the mechanisms involved in inducing responses to microbial or plant stimuli (Yergeau et al., 2014). Similarly, 13C-labeled CO_2_ enrichment is combined with metatranscriptomics to study the response of microorganisms to plant exudates released in the rhizosphere and to better understand the plant-microbiome relation (Haichar et al., 2016). Exoproteins are more stable in the environment than RNA, which has short turnover times, reducing the robustness and simplicity of sampling efficiency and making sampling more prone to errors (Prosser, 2015). Metaproteomics also enables an intriguing possibility of studying metabolic activities, as it gives the profiles of expressed proteins (Heyer et al., 2015). The ecologically important proteins for nutrient uptake and microbial–host and microbial–microbial relationships (e.g., transporter systems and extracellular hydrolytic enzymes) are enriched by exometaproteomics or exoproteomics (Lidbury et al., 2016). However, the need for enough starting material (up to 100 g of soil) (Johnson-Rollings et al., 2014), accurate peptide profiles, and adequate computational power limit the applications of metaproteomics (Muth et al., 2016). These might be the reasons that restrict the use of meta(exo)proteomics in rhizosphere research.

### Omics approaches to unveil plant-associated microbiota

Recently, the advent of omics tools, gene-editing techniques, and sequencing technology has allowed us to unravel the entangled webs of plant-microbes interactions, enhancing plant fitness and tolerance to biotic and abiotic challenges. Genomics is an effective tool for studying and predicting the interactions of microbes and plants and developing pathogen stress tolerance in plants (Frantzeskakis et al., 2020).

High genetic variability in the soil microbiome can be confirmed by multiple sequencing methodologies, such as prokaryotic16S, fungal ITS (internal transcribed spacer regions), and/or metagenomic analysis. Describing who is associated with the plant is relevant to unveiling their functions, so these microorganisms can become the extended genome partner of the host (Berendsen et al., 2012). More reports on genome engineering, gene editing, and advanced plant–microbe interaction technologies have been discussed (Frantzeskakis et al., 2019; Sharma et al., 2020). The microbiome composition can be altered by environmental factors such as soil conditions and temperature. However, plant biochemistry and the immune system also play key roles in determining the variability of the microbiome (Turner et al., 2013). Although plants bring beneficial microorganisms, such as PGPR and disease-suppressing microorganisms, it has been evident that they can also bring phytopathogens as well as human pathogenic bacteria. These harmful bacteria may enter the food chain, can cause plant disease, and can alter the entire microbiome composition (Gorshkov et al., 2020). Therefore, tools such as metagenomics, for example, offer a promising strategy to diagnose these phytopathogens (Chiu and Miller, 2019). Currently, nanopore sequencing using Oxford Nanopore Technologies (ONT) is the most encouraging technology for the identification of pathogens by metagenome sequencing (Jain et al., 2016). It is fast and is a direct sequencing method requiring no amplification step. It can be used even if we lack any prior knowledge of pathogens, as it can directly detect and identify all pathogens except RNA viruses. Moreover, it can also reconstruct the functional pathways in the microbiome and can foresee its composition. A high error rate limits the use of ONT (Rang et al., 2018). Therefore, it can be combined with Illumina technologies to enhance the sequence assembly quality (Sevim et al., 2019). MinON™ has already been used for metagenomics sequencing of bacterial, fungal and viral pathogens on several crops (Jongman et al., 2020; Mechan Llontop et al., 2020). Low sequencing cost and high quality suggest that direct sequencing is likely to be the future of metagenomics (Ciuffreda et al., 2021). An increasing number of propositions are becoming feasible because of the expanding information in metagenomics. It was first proposed that the initial molecular assessment of the soil and soil microbiome could help in the improvement of agricultural treatments (Schlaeppi and Bulgarelli, 2015). Conversely, the complimentary response of the host toward beneficial microbes should also be a part of the engineering program because the host is also involved in bringing the interaction. It would enable the plant cultivars to interact efficaciously with natural as well as acquired microorganisms (Bulgarelli et al., 2013). The drawback of genomic analysis is that it does not provide knowledge about the functional states of biological objects; therefore, a metagenomics approach can be used in combination with a transcriptomic approach to evaluate key traits in plant-microbiome interactions.

Next Generation Sequencing (NGS)-based transcriptomics is another approach used to unravel the molecular mechanisms involved in plant–microbiome interactions. It is usually applied in plant pathology and stress studies. It reveals the physiological response of plants to pathogens and characterizes the signaling events taking place in the rhizosphere. Although we can predict community function from multi-omics data alone to some extent, validation of interactions requires the complementary work with cultured isolates that can be interrogated in the laboratory (Terekhov et al., 2018; Kehe et al., 2019). For example, the resistance of barley to *Blumeria graminis* by the gene network has been uncovered by NGS (Li et al., 2020). It also revealed the underlying mechanism of resistance against *Pectobacterium atrosepticum* (Tsers et al., 2020). Moreover, the characteristic interactions between Phytophthora infestans and potato plants have been revealed by gene expression patterns or NGS (Duan et al., 2020). NGS can also be used to study plant interactions with non-infectious microbes and plant responses to abiotic stresses. For example, the tolerance of tomato to hypoxia (Safavi-Rizi et al., 2020), changes in the gene expression pattern of orchard grass due to short-term flooding (Qiao et al., 2020), and gene expression changes in Arabidopsis because of high ultraviolet stress (Huang et al., 2019) have been revealed by NGS. However, the vast data profiles generated by NGS are too enormous to be efficaciously translated into simple language. This makes the interpretation of NGS transcriptomic data difficult for higher plants (Murat et al., 2012). Moreover, in most cases, the expression level is not restricted to a single gene (Das et al., 2020). Therefore, the focus of transcriptomic studies has shifted from the individual gene level to the gene set level. Significant impact of anthropogenic activities on the plant microbiome.

**FIGURE 3:**
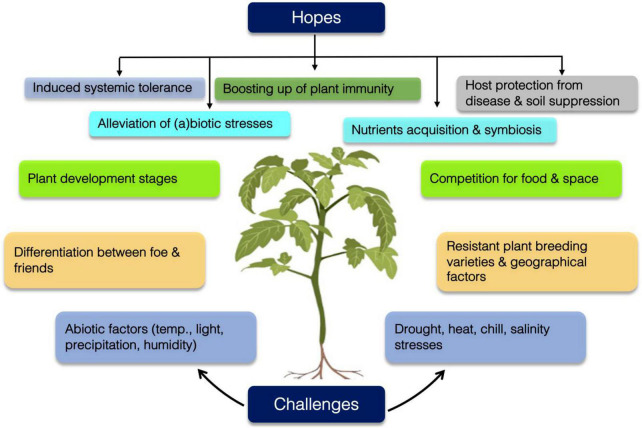
Plant Microbiome provides key functions for plant health and its protection. Plant microbiome offers vital services for plant health. It facilitates biogeochemical cycling of plant nutrients, assist plant growth under biotic and abiotic conditions, induces systemic acquired resistance (SAR) and induces systemic resistance (ISR) in plant against plant pathogen. Inversely, Plant microbiome synchronously encounters biotic and abiotic stresses which are the substantial drivers that influence or alter microbiome diversity and functionality.

Over the past few decades, industrialization and urbanization have caused an increase in carbon dioxide and temperature, which affect the climate globally. These changes cause erratic events worldwide, such as a decrease in moisture level, an increase in temperature, excessive greenhouse gas emissions, and an increase in snowfall and rainfall. Climate change, range shift and urbanization are key factors that affect plant microbial interactions in the rhizosphere (Figure 3). Soil microbial community determines the soil, and plant health and prerequisite for external constraints. Soil microbial ecosystem functions and diversity are significantly influenced by anthropogenic activities These activities produce a diverse array of hazardous substances including pesticides, heavy metals (Ma et al., 2022a) and organic pollutants and put tremendous pressure on soil microbiomes. Heavy metals notoriously imbalance the microbial population, diversity and seriously decline their activities (Abdu et al., 2017; Fajardo et al., 2019; Li et al., 2020).

### Climate change

Abrupt changes in climate and weather patterns have become a global dilemma among researchers and farmers (Amna et al., 2021; Wahab et al., 2022). Anthropogenic activities such as global warming, deforestation, the greenhouse effect, and urbanization have made these climate changes inevitable. Excessive fertilizer and pesticide use, livestock farming, nitrous oxide emissions, and fossil fuel combustion are the other contributors to climate change. The development of plants is affected by different climatic factors, such as CO_2_ levels in the atmosphere, temperature (Saeed et al., 2022), drought (Wahab et al., 2022), salinity (Mehmood et al., 2021a; Hussain et al., 2022), heavy metals (Ma et al., 2022b; Nawaz et al., 2022), and rainfall patterns. However, the impact of climate change on the variety of microfauna is also worthy of attention because microorganisms are also influenced by these changes as they perform carbon and nutrient cycling. Abrupt changes in climate can disrupt the microbial population above and below ground and can have a negative impact on plant development. For example, global warming affects microbial respiration and therefore directly alters the microbial composition (Classen et al., 2015). Temperature plays a key role in defining the microbial community of plants and is also decisive in plant phenological characteristics and development (Kashyap et al., 2017). In the past few decades, emissions of greenhouse gases (CO_2_, CH_4_, water vapor, etc.) due to rapid urbanization and industrialization has elevated the temperature. According to Compant et al. (2010), the average temperature is expected to rise by 1.8–3.6°C by 2100, which would lead to water scarcity and droughts (Farooq et al., 2022). Several studies have been performed to describe the effects of elevated temperature on plant morphology (Chen et al., 2021). Similarly, elevated temperature also influences the activities and composition of microorganisms in the rhizosphere. An increase in temperature increases the growth rate of microorganisms with altered respiration (Figure 3; Classen et al., 2015). Karhu et al. (2014) reported an exponential increase in soil respiration with increasing temperature. Additionally, organic matter utilization by microorganisms is also dependent on temperature (Frey et al., 2013). Temperature alterations are also correlated with the pathogenicity of microbes. Increased temperature increases the growth of *Glomus mossae* and *Glomus intraradices* (Monz et al., 1994). Disease incidences in plants by certain seed-borne microbes that degrade cell walls and *Pectobacterium atrosepticum* causing soft rot can be increased by an increase in temperature (Hasegawa et al., 2005). Drought conditions pose a threat to plant carbohydrate exchange and nutrient uptake in the rhizosphere by AFM (Newsham et al., 1995). In mountainous soil, the warming effect is amplified when heat waves combined with elevated temperature increase the C and N cycling of microorganisms (Donhauser et al., 2021). However, other factors, such as UV radiation and moisture, also affect microbial communities. AFM cannot colonize plants under drought conditions (Staddon et al., 2004). The bacterial population is also reduced in the rhizosphere of sorghum roots under drought conditions (Xu et al., 2018c).

The allocation of carbon in the rhizosphere is regulated by atmospheric carbon dioxide. Therefore, atmospheric CO_2_ regulates the root exudate composition in soil, which defines the microbial community in the rhizosphere (Williams et al., 2018). Microorganisms are the key factors in the net exchange of carbon in soil. They perform this function in various ways by altering the nutrient status of the soil, forming symbiotic or pathogenic interactions with plants, respiration and organic matter decomposition. Therefore, high levels of CO_2_ can alter the microbial population directly or indirectly by altering plant physiology and metabolism. Elevated CO_2_ levels alter the root exudate composition and nutrient availability in soil (Compant et al., 2010). Some fungi have the potential to assimilate more carbon than bacteria; therefore, they can store carbon than mobilization. Thus, the microbial population in soil is stimulated by excessive emission of carbon by roots. This microbial propagation eventually reduces nitrogen availability for plants because of nitrogen immobilization in the soil. Soil respiration is also increased by elevated CO_2_ levels. Microorganisms respond differently to elevated CO_2_ levels in soil. No significant effect was observed by Gavito et al. (2000) in AMF of *Pisum sativum* with an increase of 700 ppm of CO_2_, while only with an increase of 7 ppm CO_2_ was an increase in mycorrhizal colonization observed by Tang et al. (2009) in Barnyard grass. The 18S RNA sequencing-based Illumina MiSeq technique revealed a significant decrease in the populations of Glomus and Claroideoglomus species after long-term CO_2_ (550 ppm) exposure in paddy fields (Panneerselvam et al., 2020). In *Pinus strobus* and *Boswellia papyrifera* plants, an increase in CO_2_ (700 ppm) concentration increased the ectomycorrhizal fungi (ECM) population (Godbold and Berntson, 1997). Similarly, a threefold increase in ECM mycelia was observed in *P. sylvestris* with an increase in CO_2_ concentration (Fransson et al., 2005). PGPB are also influenced by the CO_2_ concentrations in the soil. Several studies have been performed to observe the effect of CO_2_ elevation on plant microbe interactions (Thakur et al., 2019; Yu and Chen, 2019; Prescott et al., 2020; Terrer et al., 2021). A threefold increase in *R. leguminosarum* was observed by Schortemeyer in the rhizosphere of white clover by an increase in CO_2_ (600 ppm) concentration (Schortemeyer et al., 1996). In addition, more efforts are required to understand the behavior of plant–microbial interactions under elevated CO_2_ levels to engineer the desired beneficial microorganisms for plant development.

### Range shifts

Human activities have introduced new species to the new habitats (Essl et al., 2011) and have caused environmental warming that expands the potential survival capabilities of these species in the habitats where they could never survive before or have contracted their habitat (Walther et al., 2009). These two reasons have triggered the shifting ranges. Plant–microbial interactions have gone through unforeseen impacts because of these range shifts. The elevation gradient provides a practical system to evaluate the effect of abiotic and biotic factors on plant–microbe interactions, microbial composition and distribution. Cobian et al. (2019) revealed that a parabolic relationship was followed by leaf fungal endophyte specialization, where specialization was maximum at the center of tree species ranges and reduced toward edges. Balsam poplars’ fungal community has higher diversity when relocated to the upper edges of the elevation gradient because they experience higher abiotic stresses (Bálint et al., 2015). Compared to fungi, leaf bacterial communities are less affected by changes in elevation gradients because fungi are more sensitive to temperature changes (Vacher et al., 2016). Along the elevation gradient, plant community dynamics also face a turn from competition to facilitation. However, a vast majority of research is required to study the positive and negative effects of elevation gradient shifts on plant-microbial interactions. Plant–soil feedback (PSF) is a mechanism by which plants influence abiotic and biotic factors in soil, and feedbacks influence their development and growth (van der Putten et al., 2016). PSF and microorganisms negatively affect native species (Bever, 2003). Previously established communities of microorganisms are reestablished by the novel soil biota through species range expansion, e.g., negative interactions develop between the soil biota and *Centaurea maculosa* in native ranges, while in North America, they develop positive interactions with microorganisms in soil (Callaway et al., 2004). The survival of non-native species in novel environments is favored by the dearth of natural enemies. A significant reduction in foliar and floral pathogens has been evident in invasive plants (Ramirez et al., 2019). In comparison, seed germination of *Acer saccharum was* reduced in soil beyond its native range limits even though the abiotic conditions were sufficient (Carteron et al., 2020). A variety of microbial interactions can influence species range shifts; however, thorough research is needed in this sector to evaluate the contrasting roles of microorganisms in driving plant range shifts.

### Urbanization

Urbanization has been a source of various airborne pollutants. The use of chemicals and micro- and macronutrients influences local vegetation, eventually altering plant-microbial interactions (Annamalai and Namasivayam, 2015). Moreover, these anthropogenic activities also have the impact of the microbial population, which has the potential to remediate air pollution. The phyllosphere communities of bacteria and fungi are distinct in rural and urban trees (Smets et al., 2016; Laforest-Lapointe et al., 2017). A 10% increase in alpha-bacteria was observed by Laforest-Lapointe et al. (2017) and Imperato et al. (2019) in urban tree leaves. Espenshade et al. (2019) also observed the impact of traffic patterns and urban density on the bacterial composition of tree leaves, which was associated with black carbon and ultrafine particulate matter. A lower diversity of fungi was observed on urban trees by Jumpponen and Jones (2010). However, a higher fungal load was observed by Imperato et al. (2019). Moreover, traffic levels also influenced the phyllosphere community of bacteria (Smets et al., 2016). These findings enable the need to better understand the elements that bring changes in the phyllosphere of urban trees and to check the varying changes that take place within microbial functions.

Recent investigations have started to generate a link between the impact of urbanization on the genetic and functional changes of the phyllosphere microbiota. For instance, a higher number of bacteria was observed in urban trees. These bacteria have genes encoding enzymes for aromatic degradation that impart PGP traits to plants (Imperato et al., 2019). Additionally, it has also been observed that hydrocarbon-degrading bacteria are selected by plants when hydrocarbon levels increase in the atmosphere (Gandolfi et al., 2017). This phenomenon is termed phytoremediation, and plant-microbial interactions play a pivotal role in efficacious phytoremediation. Endophytes can remediate soil and water contaminants and promote the growth of plants (Siciliano et al., 2001; Mukhtar et al., 2018). Soil contaminants increase the prevalence of catabolic genes in endophytes, and this phenomenon can be artificially introduced in bacteria. The introduction of toluene-degrading genes in endophytic bacteria can enhance toluene degradation in soil, thus reducing phytotoxicity and toluene evapotranspiration through the leaves by up to 70% (Barac et al., 2004). A number of studies have been performed to evaluate the contaminant-degrading capabilities of bacteria (Hong et al., 2018; Undugoda et al., 2018; Ben-Israel, 2020). However, the true potential of microorganisms and plants in degrading air and soil pollutants has yet to be discovered. In addition, these findings suggest that we need to determine the influence of urbanization on plant-microbial interactions if we want to engineer the microbiome of plants.

## Conclusion

Chemical fertilizers and pesticides have been used for a long time among agricultural platforms. The goal of using such sources is to attain better crop production as per the demand of the growing human population. Excessive implementation of these chemical means may not be an acceptable choice for sustainable ecosystems. In such a way, this review, in addition to unveiling the complexities of the plant-microbiome interactions, as well as the wide possibilities to manipulate them under stressful conditions, has unraveled vital factors that are relevant to generate sustainable agriculture. Therefore, the engineering of the microbiome is a highly fundamental approach dedicated to the betterment of the health, growth and functions of plants. Studies aiming to grasp this interplay at the community level can enhance the understanding of factors that control the microbiome assemblage with its relevant feedback to a host plant. Such goals are obtainable with the support of modern tools such as “omics,” yet combining such an innovative approach with additional efforts in rhizosphere microbiome engineering can interestingly provide new insights. Similarly, an optimized phytomicrobiome meta-organism may result in a sustainable ecosystem with better agricultural production and can similarly diminish greenhouse gas emissions and soil pollution. As the microbes in the rhizosphere are scarcely investigated, further efforts are required to monitor and engineer the arrangement and activities of this microbiome. A large body of research covered the various aspects of phytomicrobiome engineering. In the last decade, massive progress has been made in plant microbiome studies but some gaps are still needed to address and fulfilled. Understanding the importance of the plant microbiome, (1) the influence of secondary metabolites of microorganisms on beneficial microbes of the plant microbiome, (2) The alteration of continuous environmental condition and their impact on the host and its associated microbial communities, (3) to investigate the ability of host plant to refrain pathogenic microbes, (4) the integration of agronomic practices with synthetic biology and their optimization and compatibility to each other.

As per demand, further elaboration can support the comprehension of the mutual association of many microbes with their host plant based on their molecular and genetic basis under any environmental constraints, which beyond can open up new avenues to advance biological and ecological practices. Future studies are directed to explore the identified gaps and, based on current knowledge, should mainly focus on classifying those biotic and abiotic factors that responsibly influence the diversity, functions and association of the microbial communities with hosts in extreme habitats. Therefore, novel findings can lead us to better understand the ecological connections between plant and underground microbes.

## Author contributions

MA, MJ, SA, FD, and BA: conceptualization. AS, Sumaira, RM, DA, and SS: data curation. AS, Sumaira, and RM: formal analysis. GS: funding acquisition. FD, RM, DA, and SS: software. MA, MJ, SA, FD, and BA: writing – original draft. MA, MJ, SA, FD, BA, AS, Sumaira, RM, DA, SS, and GS: writing – review and editing. All the authors have reviewed, edited, and approved the manuscript before submission.
